# Supplementary material for: Original plant traceability of Dendrobium species using multi-spectroscopy fusion and mathematical models
Source: R Soc Open Sci. 2019 May 22;6(5):190399. doi: 10.1098/rsos.190399 (PMC6549973; doi:10.1098/rsos.190399)

**Original Plant Traceability of *Dendrobium* Species Using Multi-Spectroscopy Fusion and Mathematical Model**

Ye Wang ^a, b^, Zhi-Tian Zuo ^b^, Heng-Yu Huang ^a*^, Yuan-Zhong Wang ^a, b*^

^a^ College of Traditional Chinese Medicine, Yunnan University of Chinese Medicine, Kunming 650500, China

^b^ Institute of Medicinal Plants, Yunnan Academy of Agricultural Sciences, Kunming 650200, China

**^*^Corresponding authors:**

**Mr Yuan-Zhong Wang**, Institute of Medicine Plants, Yunnan Academy of Agricultural Science, 2238, Beijing Road, Panlong District, Kunming 650200, China. Tel: +86 871-65033575, Fax: +86 871-65033441, E-mail: boletus@126.com (Yuan-Zhong Wang) and **Prof. Heng-Yu Huang**, College of Traditional Chinese Medicine, Yunnan University of Chinese Medicine, 1076, Yuhua Road, Chenggong District, Kunming 650500, China. Tel: +86 871-6503-3564, Fax: 871-6503-3564, E-mail: hhyhhy96@163.com

Table S1 Vote results of random forest in calibration set before selection of important variables.

|  | 1 | 2 | 3 | 4 | 5 | 6 | 7 | 8 | 9 | 10 | 11 | 12 |
| --- | --- | --- | --- | --- | --- | --- | --- | --- | --- | --- | --- | --- |
| 1 | **0.464** | 0.1071 | 0.1071 | 0.0714 | 0.1071 | 0 | 0 | 0 | 0 | 0.1429 | 0 | 0 |
| 2 | **0.8** | 0.2 | 0 | 0 | 0 | 0 | 0 | 0 | 0 | 0 | 0 | 0 |
| 3 | **0.826** | 0.1304 | 0.0435 | 0 | 0 | 0 | 0 | 0 | 0 | 0 | 0 | 0 |
| 4 | **0.765** | 0.0588 | 0 | 0 | 0 | 0 | 0.0588 | 0 | 0.0588 | 0 | 0.0588 | 0 |
| 5 | **0.5** | 0.1875 | 0 | 0 | 0.125 | 0 | 0.0625 | 0 | 0 | 0.0625 | 0.0625 | 0 |
| 6 | **0.647** | 0.0588 | 0.0588 | 0 | 0 | 0 | 0 | 0.1765 | 0 | 0.0588 | 0 | 0 |
| 7 | **0.957** | 0 | 0 | 0 | 0 | 0 | 0 | 0 | 0 | 0.0435 | 0 | 0 |
| 8 | 0 | **0.263** | 0 | 0.0526 | 0.2105 | 0.0526 | 0 | 0.0526 | 0 | 0.1579 | 0.2105 | 0 |
| 9 | 0.2632 | **0.632** | 0.0526 | 0.0526 | 0 | 0 | 0 | 0 | 0 | 0 | 0 | 0 |
| 10 | 0.4348 | **0.522** | 0.0435 | 0 | 0 | 0 | 0 | 0 | 0 | 0 | 0 | 0 |
| 11 | 0.1429 | **0.643** | 0 | 0 | 0.1429 | 0.0714 | 0 | 0 | 0 | 0 | 0 | 0 |
| 12 | 0.1429 | **0.667** | 0 | 0.0476 | 0 | 0 | 0.0952 | 0 | 0 | 0 | 0 | 0.0476 |
| 13 | 0.0769 | **0.846** | 0 | 0 | 0.0769 | c0 | 0 | 0 | 0 | 0 | 0 | 0 |
| 14 | **0.333** | **0.333** | 0.1333 | 0 | 0 | 0 | 0.0667 | 0.0667 | 0 | 0.0667 | 0 | 0 |
| 15 | 0 | 0 | **0.722** | 0 | 0 | 0.1111 | 0 | 0.1111 | 0 | 0 | 0.0556 | 0 |
| 16 | 0 | 0 | **0.55** | 0.1 | 0 | 0.05 | 0.1 | 0.05 | 0 | 0.15 | 0 | 0 |
| 17 | 0.0435 | 0 | **0.739** | 0.0435 | 0 | 0.0435 | 0 | 0 | 0.087 | 0 | 0.0435 | 0 |
| 18 | 0 | 0 | **0.684** | 0.0526 | 0 | 0 | 0 | 0.0526 | 0 | 0.1579 | 0.0526 | 0 |
| 19 | 0 | 0 | **0.875** | 0 | 0 | 0.0417 | 0 | 0.0417 | 0 | 0 | 0.0417 | 0 |
| 20 | 0.05 | 0 | **0.55** | 0.05 | 0.05 | 0 | 0.1 | 0.2 | 0 | 0 | 0 | 0 |
| 21 | 0 | 0 | 0.0526 | **0.737** | 0.0526 | 0 | 0.0526 | 0 | 0.0526 | 0.0526 | 0 | 0 |
| 22 | 0 | 0 | 0.0435 | **0.739** | 0 | 0 | 0.0435 | 0.0435 | 0.087 | 0.0435 | 0 | 0 |
| 23 | 0 | 0 | 0.0625 | **0.563** | 0.125 | 0 | 0.0625 | 0.1875 | 0 | 0 | 0 | 0 |
| 24 | 0 | 0 | 0 | **0.815** | 0 | 0.037 | 0.037 | 0.0741 | 0.037 | 0 | 0 | 0 |
| 25 | 0 | 0.0526 | 0 | **0.737** | 0 | 0 | 0 | 0.1053 | 0.1053 | 0 | 0 | 0 |
| 26 | 0 | 0 | 0.1 | **0.5** | 0.05 | 0.05 | 0.15 | 0.1 | 0 | 0.05 | 0 | 0 |
| 27 | 0 | 0 | 0.0435 | **0.826** | 0.0435 | 0 | 0 | 0.087 | 0 | 0 | 0 | 0 |
| 28 | 0.0556 | 0 | 0 | 0 | **0.611** | 0.1111 | 0 | 0.0556 | 0.1111 | 0.0556 | 0 | 0 |
| 29 | 0 | 0.0667 | 0 | 0.1333 | **0.333** | 0.2667 | 0 | 0 | 0 | 0 | 0.1333 | 0.0667 |
| 30 | 0 | 0.0588 | 0.0588 | 0 | **0.412** | 0.0588 | 0 | 0.2353 | 0.0588 | 0.1176 | 0 | 0 |
| 31 | 0 | 0 | 0.125 | 0 | **0.5** | 0.1875 | 0.0625 | 0 | 0 | 0.0625 | 0.0625 | 0 |
| 32 | 0 | 0.0526 | 0.0526 | 0.0526 | **0.632** | 0.1053 | 0 | 0.0526 | 0.0526 | 0 | 0 | 0 |
| 33 | 0.0526 | 0.1053 | 0 | 0 | **0.263** | 0.1579 | 0.0526 | 0.1053 | 0 | 0.2105 | 0 | 0.0526 |
| 34 | 0 | 0 | 0.0556 | 0.1111 | **0.5** | 0 | 0.1667 | 0 | 0.1111 | 0 | 0 | 0.0556 |
| 35 | 0 | 0 | 0.0455 | 0 | 0.2727 | **0.273** | 0 | 0.1818 | 0.0455 | 0.1364 | 0.0455 | 0 |
| 36 | 0 | 0 | 0.0556 | 0.1111 | 0.1111 | **0.611** | 0.0556 | 0.0556 | 0 | 0 | 0 | 0 |
| 37 | 0 | 0 | 0 | 0.0909 | 0.0909 | **0.727** | 0 | 0.0455 | 0.0455 | 0 | 0 | 0 |
| 38 | 0.0455 | 0 | 0 | 0 | 0 | **0.727** | 0.0455 | 0.0455 | 0 | 0.0909 | 0.0455 | 0 |
| 39 | 0 | 0 | 0.0556 | 0 | 0 | **0.722** | 0.0556 | 0.0556 | 0.0556 | 0.0556 | 0 | 0 |
| 40 | 0 | 0 | 0 | 0 | 0 | **0.714** | 0.0714 | 0.0714 | 0.0714 | 0.0714 | 0 | 0 |
| 41 | 0 | 0 | 0.0526 | 0.1053 | 0 | **0.737** | 0 | 0.0526 | 0 | 0 | 0.0526 | 0 |
| 42 | 0 | 0 | 0.0714 | 0 | 0 | 0.0357 | **0.821** | 0 | 0.0357 | 0 | 0.0357 | 0 |
| 43 | 0 | 0 | 0.0476 | 0 | 0.0476 | 0 | **0.762** | 0 | 0 | 0.1429 | 0 | 0 |
| 44 | 0 | 0 | 0.0455 | 0.0909 | 0 | 0.0455 | **0.636** | 0.1818 | 0 | 0 | 0 | 0 |
| 45 | 0.0556 | 0 | 0 | 0.1111 | 0 | 0 | **0.778** | 0 | 0 | 0.0556 | 0 | 0 |
| 46 | 0 | 0 | 0 | 0 | 0 | 0.1 | **0.55** | 0.05 | 0.05 | 0.15 | 0 | 0.1 |
| 47 | 0.0625 | 0 | 0 | 0 | 0.0625 | 0 | **0.813** | 0 | 0 | 0.0625 | 0 | 0 |
| 48 | 0.0476 | 0 | 0 | 0 | 0.0476 | 0.0476 | **0.619** | 0.0952 | 0 | 0.0952 | 0.0476 | 0 |
| 49 | 0.0455 | 0 | 0.1364 | 0 | 0.1364 | 0 | 0.0909 | **0.364** | 0 | 0.2273 | 0 | 0 |
| 50 | 0.0952 | 0.0476 | 0.0952 | 0 | 0 | 0.0952 | 0 | **0.667** | 0 | 0 | 0 | 0 |
| 51 | 0.1111 | 0 | 0.1481 | 0 | 0.1111 | 0.1111 | 0.037 | **0.481** | 0 | 0 | 0 | 0 |
| 52 | 0.0714 | 0 | 0 | 0 | 0.1429 | 0.0714 | 0.1429 | **0.357** | 0 | 0.1429 | 0.0714 | 0 |
| 53 | 0 | 0 | 0.1 | 0 | 0.05 | 0.05 | 0 | **0.75** | 0 | 0.05 | 0 | 0 |
| 54 | 0.0588 | 0 | 0 | 0 | 0.0588 | 0.1176 | 0 | **0.588** | 0 | 0.0588 | 0.1176 | 0 |
| 55 | 0.0526 | 0 | 0.0526 | 0 | 0 | 0 | 0 | **0.684** | 0.1053 | 0.1053 | 0 | 0 |
| 56 | 0.0476 | 0.0476 | 0 | 0 | 0 | 0 | 0 | 0 | **0.762** | 0.1429 | 0 | 0 |
| 57 | 0 | 0 | 0.0417 | 0.125 | 0 | 0.0417 | 0.0833 | 0.0417 | **0.667** | 0 | 0 | 0 |
| 58 | 0 | 0.0385 | 0.1154 | 0.0385 | 0 | 0.0385 | 0 | 0.0769 | **0.538** | 0.1538 | 0 | 0 |
| 59 | 0 | 0 | 0 | 0.1154 | 0 | 0 | 0.0769 | 0.0385 | **0.731** | 0.0385 | 0 | 0 |
| 60 | 0 | 0 | 0 | 0 | 0 | 0.1333 | 0.0667 | 0 | **0.733** | 0.0667 | 0 | 0 |
| 61 | 0 | 0 | 0 | 0 | 0 | 0.0435 | 0.0435 | 0.0435 | **0.783** | 0.087 | 0 | 0 |
| 62 | 0 | 0 | 0 | 0 | 0 | 0.087 | 0.0435 | 0.087 | **0.739** | 0.0435 | 0 | 0 |
| 63 | 0 | 0 | 0.0476 | 0.0476 | **0.333** | 0.0476 | 0.0476 | 0.0476 | 0.0476 | **0.333** | 0.0476 | 0 |
| 64 | 0 | 0 | 0.0833 | 0.0417 | 0.0833 | 0.0417 | 0.0417 | 0 | 0 | **0.542** | 0.125 | 0.0417 |
| 65 | 0 | 0 | 0.0455 | 0 | 0.0455 | 0 | 0 | 0 | 0.0455 | **0.864** | 0 | 0 |
| 66 | 0.0385 | 0.0385 | 0.0769 | 0.0769 | 0 | 0.0385 | 0 | 0.0769 | 0 | **0.615** | 0.0385 | 0 |
| 67 | 0 | 0 | 0.0455 | 0.0455 | 0 | 0.0455 | 0.0455 | 0 | 0.0455 | **0.727** | 0.0455 | 0 |
| 68 | 0 | 0 | 0 | 0.05 | 0.05 | 0 | 0 | 0.1 | 0.1 | **0.65** | 0.05 | 0 |
| 69 | 0.037 | 0.037 | 0 | 0 | 0.0741 | 0.0741 | 0 | 0 | 0 | **0.704** | 0.0741 | 0 |
| 70 | 0 | 0.15 | 0.05 | 0 | 0.1 | 0 | 0.05 | 0.05 | 0 | 0.1 | **0.5** | 0 |
| 71 | 0 | 0 | 0 | 0 | 0.0952 | 0.0476 | 0.0952 | 0 | 0 | 0.1905 | **0.524** | 0.0476 |
| 72 | 0 | 0.0435 | 0.0435 | 0.0435 | 0 | 0 | 0.0435 | 0 | 0.087 | 0.0435 | **0.696** | 0 |
| 73 | 0 | 0 | 0 | 0 | 0 | 0 | 0.0667 | 0.0667 | 0 | 0.1333 | **0.733** | 0 |
| 74 | 0 | 0 | 0.05 | 0.05 | 0 | 0 | 0.05 | 0.05 | 0.1 | 0 | **0.65** | 0.05 |
| 75 | 0 | 0.04 | 0 | 0 | 0 | 0 | 0.04 | 0.04 | 0 | 0 | **0.88** | 0 |
| 76 | 0 | 0 | 0 | 0 | 0 | 0.125 | 0 | 0 | 0 | 0.0625 | **0.75** | 0.0625 |
| 77 | 0 | 0.0769 | 0 | 0 | 0 | 0.0385 | 0.0769 | 0.0769 | 0 | 0 | 0.0769 | **0.654** |
| 78 | 0.0952 | 0 | 0 | 0 | 0 | 0.0476 | 0.0476 | 0 | 0.0476 | 0 | 0.2381 | **0.524** |
| 79 | 0.05 | 0.05 | 0 | 0.05 | 0.05 | 0.05 | 0 | 0 | 0 | 0 | 0.05 | **0.7** |
| 80 | 0.0625 | 0 | 0 | 0.0625 | 0.0625 | 0 | 0 | 0 | 0.0625 | 0 | 0.125 | **0.625** |
| 81 | 0 | 0 | 0 | 0 | 0 | 0 | 0.0476 | 0.0476 | 0.0476 | 0 | 0.1905 | **0.667** |
| 82 | 0.0417 | 0.0417 | 0 | 0 | 0.0417 | 0 | 0 | 0 | 0.0417 | 0.0417 | 0.0833 | **0.708** |
| 83 | 0.0476 | 0.0476 | 0 | 0.0476 | 0.0952 | 0 | 0 | 0 | 0 | 0 | 0 | **0.762** |

Table S2 Vote results of random forest in validation set using low-level fusion strategy.

|  | 1 | 2 | 3 | 4 | 5 | 6 | 7 | 8 | 9 | 10 | 11 | 12 |
| --- | --- | --- | --- | --- | --- | --- | --- | --- | --- | --- | --- | --- |
| 1 | **0.696** | 0.0536 | 0.0179 | 0 | 0.0536 | 0 | 0.0357 | 0.0179 | 0.0179 | 0.0893 | 0 | 0.0179 |
| 2 | **0.911** | 0.0357 | 0.0536 | 0 | 0 | 0 | 0 | 0 | 0 | 0 | 0 | 0 |
| 3 | **0.821** | 0.0714 | 0.0179 | 0 | 0.0179 | 0 | 0.0179 | 0.0179 | 0 | 0.0357 | 0 | 0 |
| 4 | 0.0357 | **0.571** | 0.0357 | 0.0357 | 0.1607 | 0.0179 | 0.0179 | 0.0179 | 0 | 0.0714 | 0.0357 | 0 |
| 5 | 0.0536 | **0.536** | 0.0536 | 0.0357 | 0.1786 | 0.0536 | 0 | 0.0179 | 0 | 0.0357 | 0.0357 | 0 |
| 6 | 0.2143 | **0.643** | 0 | 0.0179 | 0.0357 | 0 | 0 | 0.0179 | 0 | 0.0179 | 0 | 0.0536 |
| 7 | 0.0179 | 0 | **0.732** | 0.0357 | 0 | 0 | 0.0357 | 0.1071 | 0.0179 | 0.0536 | 0 | 0 |
| 8 | 0 | 0 | **0.786** | 0.0357 | 0 | 0.0179 | 0.0179 | 0.0357 | 0.0357 | 0.0714 | 0 | 0 |
| 9 | 0.0179 | 0 | **0.75** | 0.0357 | 0 | 0.0179 | 0.0714 | 0.0536 | 0.0179 | 0.0179 | 0.0179 | 0 |
| 10 | 0 | 0.0179 | 0.0179 | **0.821** | 0.0714 | 0.0179 | 0 | 0.0536 | 0 | 0 | 0 | 0 |
| 11 | 0 | 0 | 0.0357 | **0.768** | 0.0893 | 0.0536 | 0 | 0.0357 | 0.0179 | 0 | 0 | 0 |
| 12 | 0 | 0.0179 | 0.0714 | **0.732** | 0.0714 | 0.0357 | 0 | 0.0714 | 0 | 0 | 0 | 0 |
| 13 | 0.1071 | 0.1071 | 0 | 0.0357 | **0.571** | 0.125 | 0.0179 | 0.0179 | 0.0179 | 0 | 0 | 0 |
| 14 | 0.0536 | 0.0536 | 0.0357 | 0.0357 | **0.571** | 0.0536 | 0.0179 | 0.0536 | 0.0179 | 0.0893 | 0.0179 | 0 |
| 15 | 0.0357 | 0.0179 | 0.0536 | 0.0536 | **0.464** | 0.1607 | 0.0179 | 0.0357 | 0 | 0.1607 | 0 | 0 |
| 16 | 0 | 0 | 0 | 0.0714 | 0.0179 | **0.821** | 0 | 0.0714 | 0 | 0 | 0 | 0.0179 |
| 17 | 0.0179 | 0 | 0.0357 | 0.0893 | 0.0357 | **0.732** | 0.0179 | 0.0536 | 0 | 0 | 0.0179 | 0 |
| 18 | 0.0179 | 0 | 0 | 0 | 0.1071 | **0.732** | 0 | 0.0536 | 0.0357 | 0.0179 | 0.0179 | 0.0179 |
| 19 | 0.0179 | 0 | 0.0357 | 0.0179 | 0.0179 | 0.0536 | **0.696** | 0.0893 | 0.0536 | 0.0179 | 0 | 0 |
| 20 | 0.0357 | 0 | 0 | 0.0179 | 0.0179 | 0 | **0.857** | 0.0714 | 0 | 0 | 0 | 0 |
| 21 | 0.0357 | 0 | 0 | 0.0179 | 0.0179 | 0 | **0.893** | 0 | 0 | 0.0357 | 0 | 0 |
| 22 | 0.0536 | 0 | 0.0714 | 0 | 0.0714 | 0.0357 | 0.0357 | **0.661** | 0 | 0.0536 | 0.0179 | 0 |
| 23 | 0 | 0 | 0 | 0.0357 | 0.0357 | 0.0357 | 0 | **0.875** | 0 | 0.0179 | 0 | 0 |
| 24 | 0.0357 | 0 | 0.0357 | 0 | 0.1429 | 0 | 0.0357 | **0.714** | 0 | 0.0357 | 0 | 0 |
| 25 | 0 | 0 | 0.0179 | 0 | 0 | 0.0357 | 0 | 0.0714 | **0.804** | 0.0714 | 0 | 0 |
| 26 | 0 | 0 | 0 | 0.0357 | 0 | 0.0357 | 0.0357 | 0 | **0.875** | 0.0179 | 0 | 0 |
| 27 | 0.0179 | 0 | 0.0179 | 0.0536 | 0.0179 | 0.0357 | 0.0179 | 0.0179 | **0.768** | 0.0536 | 0 | 0 |
| 28 | 0.0179 | 0 | 0.0357 | 0.0536 | 0.0536 | 0 | 0.0714 | 0.0179 | 0.1964 | **0.464** | 0.0893 | 0 |
| 29 | 0.0179 | 0.0179 | 0.0179 | 0.0179 | 0.1071 | 0.0536 | 0.0179 | 0.0179 | 0 | **0.732** | 0 | 0 |
| 30 | 0.0357 | 0.0179 | 0.0357 | 0.0179 | 0.0357 | 0 | 0.0179 | 0.0357 | 0 | **0.714** | 0.0714 | 0.0179 |
| 31 | 0.0179 | 0.0179 | 0 | 0 | 0.0357 | 0 | 0.0179 | 0.0179 | 0.0179 | 0.0179 | **0.839** | 0.0179 |
| 32 | 0 | 0.0179 | 0.0357 | 0 | 0.0357 | 0.0179 | 0 | 0.0357 | 0 | 0.0536 | **0.804** | 0 |
| 33 | 0 | 0.0179 | 0.0357 | 0.0179 | 0.0179 | 0 | 0 | 0 | 0.0179 | 0.0357 | **0.804** | 0.0536 |
| 34 | 0 | 0.0536 | 0 | 0.0179 | 0 | 0.0357 | 0.0536 | 0 | 0.0179 | 0 | 0.0536 | **0.768** |
| 35 | 0.0536 | 0.0536 | 0 | 0 | 0.0179 | 0 | 0.0179 | 0 | 0.0179 | 0 | 0.0536 | **0.786** |
| 36 | 0.0357 | 0.0536 | 0 | 0 | 0.0357 | 0 | 0.0536 | 0 | 0.0179 | 0 | 0.0179 | **0.786** |

Table S3 Confusion matrix of random forest in calibration set after selection of important variables.

|  | 1 | 2 | 3 | 4 | 5 | 6 | 7 | 8 | 9 | 10 | 11 | 12 |
| --- | --- | --- | --- | --- | --- | --- | --- | --- | --- | --- | --- | --- |
| 1 | 6 | 1 | 0 | 0 | 0 | 0 | 0 | 0 | 0 | 0 | 0 | 0 |
| 2 | 3 | 1 | 3 | 0 | 0 | 0 | 0 | 0 | 0 | 0 | 0 | 0 |
| 3 | 0 | 1 | 5 | 0 | 0 | 0 | 0 | 0 | 0 | 0 | 0 | 0 |
| 4 | 0 | 0 | 0 | 7 | 0 | 0 | 0 | 0 | 0 | 0 | 0 | 0 |
| 5 | 0 | 0 | 0 | 2 | 5 | 0 | 0 | 0 | 0 | 0 | 0 | 0 |
| 6 | 0 | 0 | 0 | 0 | 5 | 0 | 2 | 0 | 0 | 0 | 0 | 0 |
| 7 | 0 | 0 | 0 | 0 | 0 | 0 | 7 | 0 | 0 | 0 | 0 | 0 |
| 8 | 0 | 0 | 0 | 0 | 0 | 0 | 1 | 6 | 0 | 0 | 0 | 0 |
| 9 | 0 | 0 | 0 | 0 | 0 | 0 | 0 | 4 | 0 | 3 | 0 | 0 |
| 10 | 0 | 0 | 0 | 0 | 0 | 0 | 0 | 0 | 1 | 6 | 0 | 0 |
| 11 | 0 | 0 | 0 | 0 | 0 | 0 | 0 | 0 | 0 | 0 | 7 | 0 |
| 12 | 0 | 0 | 0 | 0 | 0 | 0 | 0 | 0 | 0 | 0 | 3 | 4 |
| SNE | 0.8571 | 0.1429 | 0.8333 | 1.0000 | 0.7143 | 0.0000 | 1.0000 | 0.8571 | 0.0000 | 0.8571 | 1.0000 | 0.5714 |
| SPE | 0.9610 | 0.9737 | 0.9610 | 0.9737 | 0.9342 | 1.0000 | 0.9605 | 0.9474 | 0.9868 | 0.9605 | 0.9605 | 1.0000 |
| PRE | 0.6667 | 0.3333 | 0.6250 | 0.7778 | 0.5000 | N | 0.7000 | 0.6000 | 0.0000 | 0.6667 | 0.7000 | 1.0000 |
| EFF | 0.9076 | 0.3730 | 0.8949 | 0.9868 | 0.8169 | 0.0000 | 0.9801 | 0.9011 | 0.0000 | 0.9074 | 0.9801 | 0.7559 |

Note: N means the parameter could be calculated.

Table S4 Vote results of random forest in calibration set using mid-level fusion strategy.

|  | 1 | 2 | 3 | 4 | 5 | 6 | 7 | 8 | 9 | 10 | 11 | 12 |
| --- | --- | --- | --- | --- | --- | --- | --- | --- | --- | --- | --- | --- |
| 1 | **0.512** | 0.0833 | 0.0119 | 0 | 0 | 0.0119 | 0 | 0.0357 | 0.0714 | 0.0476 | 0.2143 | 0.0119 |
| 2 | **0.359** | 0.2564 | 0 | 0.0513 | 0.0128 | 0.0769 | 0.0385 | 0.0641 | 0.0769 | 0.0256 | 0.0256 | 0.0128 |
| 3 | **0.747** | 0.046 | 0.0115 | 0.0115 | 0 | 0.069 | 0 | 0.092 | 0 | 0 | 0 | 0.023 |
| 4 | **0.521** | 0.1268 | 0.0282 | 0 | 0.0423 | 0.0704 | 0 | 0.0986 | 0.0141 | 0.0141 | 0.0141 | 0.0704 |
| 5 | **0.605** | 0.0526 | 0.0263 | 0 | 0.0132 | 0.0526 | 0 | 0.1184 | 0 | 0.0789 | 0.0263 | 0.0263 |
| 6 | **0.525** | 0.1375 | 0.05 | 0.025 | 0.075 | 0 | 0.0125 | 0.0125 | 0.025 | 0.075 | 0.05 | 0.0125 |
| 7 | **0.578** | 0.1687 | 0 | 0 | 0.0361 | 0.0482 | 0.012 | 0.1084 | 0 | 0.012 | 0.012 | 0.0241 |
| 8 | 0.2143 | **0.452** | 0.0119 | 0.0119 | 0.0952 | 0.0357 | 0.0714 | 0.0357 | 0.0238 | 0.0119 | 0 | 0.0357 |
| 9 | 0.1923 | **0.487** | 0.0513 | 0.0128 | 0.0641 | 0.0385 | 0.0256 | 0 | 0.0128 | 0.0769 | 0.0256 | 0.0128 |
| 10 | 0.2727 | **0.284** | 0.0227 | 0.0568 | 0.0227 | 0.0114 | 0.0114 | 0.0341 | 0.1591 | 0.0682 | 0 | 0.0568 |
| 11 | 0.0625 | **0.263** | 0.0625 | 0.0125 | 0.2375 | 0.025 | 0.1625 | 0.0875 | 0 | 0.0125 | 0.025 | 0.05 |
| 12 | 0.0824 | **0.6** | 0.0471 | 0.0353 | 0.0471 | 0.0118 | 0.0235 | 0.0353 | 0.0118 | 0 | 0.0706 | 0.0353 |
| 13 | 0.25 | **0.38** | 0.0543 | 0.0217 | 0.0761 | 0.0109 | 0.0109 | 0.0543 | 0.0109 | 0.0652 | 0.0326 | 0.0326 |
| 14 | 0.1728 | **0.296** | 0.1481 | 0.0247 | 0.1481 | 0.037 | 0.0123 | 0.0123 | 0.0123 | 0.0123 | 0.0741 | 0.0494 |
| 15 | 0.0122 | 0.0976 | **0.646** | 0.0366 | 0.0366 | 0 | 0 | 0.0244 | 0.0244 | 0.0854 | 0.0366 | 0 |
| 16 | 0.0263 | 0.0395 | **0.711** | 0.0263 | 0.0658 | 0 | 0.0526 | 0.0132 | 0.0263 | 0.0263 | 0.0132 | 0 |
| 17 | 0 | 0.0778 | **0.689** | 0.0556 | 0 | 0.0444 | 0.0222 | 0.0222 | 0 | 0.0667 | 0 | 0.0222 |
| 18 | 0.0128 | 0.0513 | **0.731** | 0.0128 | 0 | 0 | 0.0385 | 0 | 0 | 0.0128 | 0.1282 | 0.0128 |
| 19 | 0 | 0.025 | **0.7** | 0 | 0.05 | 0.0125 | 0.075 | 0.0375 | 0.025 | 0.0625 | 0 | 0.0125 |
| 20 | 0 | 0.0233 | **0.721** | 0.0116 | 0.1047 | 0 | 0.0233 | 0 | 0.0233 | 0.0349 | 0.0581 | 0 |
| 21 | 0.0112 | 0.0337 | 0 | **0.775** | 0.0787 | 0.0449 | 0.0112 | 0.0225 | 0 | 0.0112 | 0 | 0.0112 |
| 22 | 0.0116 | 0.0116 | 0.0814 | **0.698** | 0.0233 | 0.0465 | 0.0116 | 0.0116 | 0.0465 | 0.0233 | 0 | 0.0349 |
| 23 | 0 | 0.0267 | 0.0133 | **0.813** | 0.0267 | 0.04 | 0.0133 | 0.0267 | 0 | 0.0267 | 0.0133 | 0 |
| 24 | 0 | 0.0488 | 0.0122 | **0.854** | 0.0244 | 0.0244 | 0 | 0.0244 | 0 | 0 | 0 | 0.0122 |
| 25 | 0 | 0.0353 | 0 | **0.871** | 0.0118 | 0.0235 | 0.0235 | 0.0118 | 0.0118 | 0 | 0.0118 | 0 |
| 26 | 0.0247 | 0.0494 | 0.0617 | **0.605** | 0.0741 | 0.0247 | 0 | 0.0494 | 0 | 0.0247 | 0.037 | 0.0494 |
| 27 | 0.0119 | 0.0952 | 0.0476 | **0.69** | 0.0357 | 0.0357 | 0.0119 | 0 | 0.0238 | 0 | 0.0357 | 0.0119 |
| 28 | 0.0263 | 0.1053 | 0 | 0.0526 | **0.382** | 0.0526 | 0.0658 | 0.1316 | 0 | 0.1053 | 0.0395 | 0.0395 |
| 29 | 0 | 0.0779 | 0.1558 | 0.0779 | **0.364** | 0.0649 | 0 | 0.2078 | 0.026 | 0.013 | 0.013 | 0 |
| 30 | 0.0119 | 0.0595 | 0.0357 | 0 | **0.655** | 0.0833 | 0.0357 | 0.0357 | 0.0119 | 0.0238 | 0.0357 | 0.0119 |
| 31 | 0.0128 | 0.0641 | 0 | 0.0641 | **0.449** | 0.1667 | 0.0385 | 0.0256 | 0.1282 | 0.0385 | 0.0128 | 0 |
| 32 | 0.0256 | 0.1282 | 0.0128 | 0.0385 | **0.41** | 0.1154 | 0.0128 | 0.0641 | 0.1026 | 0.0256 | 0.0385 | 0.0256 |
| 33 | 0.026 | 0.1429 | 0 | 0.013 | **0.403** | 0.2468 | 0 | 0.0519 | 0.013 | 0.0519 | 0.013 | 0.039 |
| 34 | 0.037 | 0.0741 | 0.0617 | 0.0617 | **0.358** | 0.0494 | 0.0864 | 0.1481 | 0.037 | 0.0247 | 0.0617 | 0 |
| 35 | 0.0241 | 0.012 | 0.012 | 0 | 0.0602 | **0.566** | 0 | 0.1687 | 0.0482 | 0.0361 | 0.0482 | 0.0241 |
| 36 | 0.0267 | 0.0267 | 0 | 0.1333 | 0.04 | **0.653** | 0.0267 | 0.0133 | 0 | 0.04 | 0.0267 | 0.0133 |
| 37 | 0 | 0 | 0.022 | 0.033 | 0.0879 | **0.429** | 0.022 | 0.1209 | 0.033 | 0.2308 | 0.022 | 0 |
| 38 | 0.0128 | 0.0128 | 0 | 0.1667 | 0.0513 | **0.615** | 0 | 0.0385 | 0.0513 | 0.0128 | 0.0128 | 0.0256 |
| 39 | 0.0114 | 0.0341 | 0 | 0.0227 | 0.0114 | **0.705** | 0.0114 | 0.1023 | 0.0795 | 0.0114 | 0 | 0.0114 |
| 40 | 0.0568 | 0.0341 | 0 | 0.1932 | 0.0682 | **0.5** | 0 | 0.0455 | 0.0682 | 0.0227 | 0 | 0.0114 |
| 41 | 0.0112 | 0.0112 | 0 | 0.0337 | 0.0674 | **0.719** | 0.0225 | 0.0787 | 0.0225 | 0.0225 | 0 | 0.0112 |
| 42 | 0 | 0.0417 | 0.0313 | 0.0104 | 0.0313 | 0 | **0.813** | 0.0208 | 0 | 0 | 0.0208 | 0.0313 |
| 43 | 0 | 0.0476 | 0.1071 | 0.0238 | 0.1071 | 0.0238 | **0.488** | 0.0357 | 0.1071 | 0.0357 | 0.0238 | 0 |
| 44 | 0.0361 | 0 | 0 | 0.0361 | 0.0241 | 0.0241 | **0.422** | 0.0361 | 0.0361 | 0.0602 | 0.241 | 0.0843 |
| 45 | 0 | 0.0349 | 0.0233 | 0.0349 | 0.0233 | 0.0116 | **0.791** | 0.0465 | 0.0116 | 0 | 0.0233 | 0 |
| 46 | 0.0274 | 0 | 0.0137 | 0.0685 | 0.0274 | 0.0411 | **0.658** | 0.0685 | 0.0274 | 0.0274 | 0 | 0.0411 |
| 47 | 0.013 | 0.039 | 0.039 | 0.013 | 0.026 | 0 | **0.662** | 0.026 | 0.039 | 0.039 | 0.0519 | 0.0519 |
| 48 | 0.0123 | 0 | 0.0247 | 0.0123 | 0.0247 | 0 | **0.84** | 0.0247 | 0.0247 | 0.0123 | 0.0123 | 0.0123 |
| 49 | 0.0127 | 0.0633 | 0.0127 | 0.0127 | 0.1646 | 0.1139 | 0.0633 | **0.253** | 0 | 0.2278 | 0 | 0.0759 |
| 50 | 0.0357 | 0.0238 | 0.1071 | 0.0238 | 0.0476 | 0.1667 | 0.0476 | **0.512** | 0.0119 | 0.0119 | 0.0119 | 0 |
| 51 | 0.0625 | 0.075 | 0.0125 | 0 | 0.0125 | 0.025 | 0.025 | **0.7** | 0 | 0.05 | 0 | 0.0375 |
| 52 | 0.0132 | 0.0132 | 0.0132 | 0.0921 | 0.0263 | 0.0395 | 0 | **0.605** | 0 | 0.1447 | 0.0132 | 0.0395 |
| 53 | 0.0241 | 0 | 0.012 | 0 | 0.012 | 0.0361 | 0.0361 | **0.855** | 0 | 0.0241 | 0 | 0 |
| 54 | 0.0388 | 0.0194 | 0.0291 | 0.0194 | 0.0194 | 0.1068 | 0.0194 | **0.437** | 0.0291 | 0.0583 | 0.2039 | 0.0194 |
| 55 | 0.2073 | 0.0732 | 0.0122 | 0 | 0.0122 | 0.0122 | 0.0122 | **0.585** | 0 | 0.0122 | 0.0244 | 0.0488 |
| 56 | 0.0513 | 0.0385 | 0.0256 | 0 | 0.0641 | 0.1154 | 0.0513 | 0.0385 | **0.372** | 0.1667 | 0.0128 | 0.0641 |
| 57 | 0 | 0 | 0 | 0 | 0.0805 | 0.0115 | 0.023 | 0.0115 | **0.816** | 0.0345 | 0.0115 | 0.0115 |
| 58 | 0 | 0 | 0 | 0 | 0.0112 | 0.0112 | 0.0112 | 0 | **0.91** | 0.0225 | 0.0112 | 0.0225 |
| 59 | 0.0119 | 0 | 0.0714 | 0.0119 | 0.0238 | 0.0357 | 0.0714 | 0.0238 | **0.655** | 0.0595 | 0.0357 | 0 |
| 60 | 0.0588 | 0.0118 | 0 | 0.0235 | 0.0118 | 0.0118 | 0.0235 | 0 | **0.824** | 0.0118 | 0.0235 | 0 |
| 61 | 0 | 0.0449 | 0 | 0 | 0.0562 | 0.0112 | 0.0337 | 0.0674 | **0.73** | 0.0449 | 0.0112 | 0 |
| 62 | 0.0506 | 0.0127 | 0 | 0 | 0.0506 | 0 | 0 | 0.0253 | **0.81** | 0.038 | 0.0127 | 0 |
| 63 | 0.0875 | 0.05 | 0.05 | 0.025 | 0.075 | 0.0375 | 0.0375 | 0.05 | 0.0375 | **0.463** | 0.0375 | 0.05 |
| 64 | 0.0349 | 0.0698 | 0.0116 | 0.0349 | 0.0814 | 0.0581 | 0.0581 | 0.0116 | 0.0349 | **0.558** | 0.0465 | 0 |
| 65 | 0.0465 | 0.0465 | 0.0349 | 0.0233 | 0.093 | 0.0581 | 0.0465 | 0.0349 | 0.0465 | **0.442** | 0.0349 | 0.093 |
| 66 | 0.0211 | 0 | 0.0105 | 0.0316 | 0.0211 | 0.0316 | 0.0105 | 0.0421 | 0.0105 | **0.779** | 0.0316 | 0.0105 |
| 67 | 0.05 | 0.05 | 0.025 | 0.0125 | 0.0625 | 0.05 | 0.0125 | 0.0375 | 0.0125 | **0.663** | 0 | 0.025 |
| 68 | 0.0114 | 0.0341 | 0.0227 | 0.0114 | 0.0909 | 0.0341 | 0.0114 | 0 | 0.0114 | **0.716** | 0.0114 | 0.0455 |
| 69 | 0.0119 | 0 | 0.0357 | 0.0119 | 0.1429 | 0.0119 | 0.0238 | 0.0238 | 0.0476 | **0.643** | 0.0357 | 0.0119 |
| 70 | 0.0449 | 0.0787 | 0.0225 | 0 | 0.0899 | 0.0112 | 0.0562 | 0.0337 | 0.0337 | 0.0674 | **0.528** | 0.0337 |
| 71 | 0.0357 | 0.0238 | 0.0476 | 0.0119 | 0.0238 | 0.2143 | 0.0476 | 0.1548 | 0 | 0.0119 | **0.417** | 0.0119 |
| 72 | 0.0111 | 0 | 0.0333 | 0 | 0.0778 | 0 | 0.0444 | 0.0222 | 0.0333 | 0.2111 | **0.544** | 0.0222 |
| 73 | 0.0222 | 0.0111 | 0.0333 | 0 | 0.0222 | 0.0333 | 0 | 0.0111 | 0.0111 | 0 | **0.822** | 0.0333 |
| 74 | 0.0366 | 0.0366 | 0 | 0.0488 | 0.0366 | 0.0244 | 0.0244 | 0 | 0.0122 | 0.0122 | **0.768** | 0 |
| 75 | 0.0698 | 0.0814 | 0 | 0 | 0.0465 | 0 | 0.0233 | 0.0465 | 0 | 0.0581 | **0.674** | 0 |
| 76 | 0.0267 | 0.0133 | 0.04 | 0 | 0.0267 | 0 | 0.0133 | 0.0267 | 0 | 0 | **0.853** | 0 |
| 77 | 0.0253 | 0.0886 | 0.0633 | 0.0633 | 0.038 | 0.1139 | 0.1646 | 0.0759 | 0 | 0.0633 | 0.0506 | **0.253** |
| 78 | 0.0562 | 0.0787 | 0.0225 | 0.0449 | 0.0674 | 0 | 0.0337 | 0 | 0.2247 | 0.0899 | 0.0112 | **0.371** |
| 79 | 0.0405 | 0.0676 | 0.0811 | 0.0811 | 0.027 | 0.0541 | 0.0946 | 0.0135 | 0.0405 | 0.0541 | 0.0676 | **0.378** |
| 80 | 0.1235 | 0.0617 | 0.0494 | 0.0247 | 0.1358 | 0.0617 | 0.0494 | 0.0988 | 0.0247 | 0.0494 | 0.0247 | **0.296** |
| 81 | 0.0115 | 0.046 | 0.069 | 0.023 | 0.0575 | 0.1034 | **0.276** | 0.0345 | 0.023 | 0.0805 | 0.0345 | 0.2414 |
| 82 | 0.0682 | 0.0341 | 0.0568 | 0.0455 | 0.0568 | 0.0341 | 0.125 | 0.0909 | 0.1136 | 0.0568 | 0.0227 | **0.295** |
| 83 | 0.0225 | 0.0112 | 0.1348 | 0.0449 | 0.0449 | 0.0225 | 0.0337 | 0.0449 | 0.0562 | 0.1685 | 0.0562 | **0.36** |

Table S5 Vote results of random forest in validation set using mid-level fusion strategy.

|  | 1 | 2 | 3 | 4 | 5 | 6 | 7 | 8 | 9 | 10 | 11 | 12 |
| --- | --- | --- | --- | --- | --- | --- | --- | --- | --- | --- | --- | --- |
| 1 | **0.734** | 0.0917 | 0 | 0.0087 | 0.0087 | 0.0306 | 0.0131 | 0.0437 | 0.0218 | 0.0131 | 0.0175 | 0.0175 |
| 2 | **0.764** | 0.0961 | 0 | 0.0044 | 0.0131 | 0.0218 | 0.0175 | 0.048 | 0.0131 | 0.0087 | 0 | 0.0131 |
| 3 | **0.817** | 0.048 | 0.0044 | 0.0044 | 0 | 0.048 | 0 | 0.0437 | 0 | 0.0044 | 0.0131 | 0.0175 |
| 4 | 0.131 | **0.607** | 0.0568 | 0.0306 | 0.048 | 0.0087 | 0.0218 | 0.0175 | 0.0044 | 0.0044 | 0.0044 | 0.0655 |
| 5 | 0.0524 | **0.594** | 0.0087 | 0.0131 | 0.1485 | 0.0218 | 0.0349 | 0.0742 | 0.0087 | 0.0044 | 0.0087 | 0.0306 |
| 6 | 0.0961 | **0.459** | 0.0087 | 0.0961 | 0.0175 | 0.0044 | 0.0044 | 0.0306 | 0.0961 | 0.0917 | 0.0611 | 0.0349 |
| 7 | 0 | 0.0131 | **0.904** | 0.0044 | 0.0175 | 0 | 0 | 0.0044 | 0.0044 | 0.0262 | 0.0175 | 0.0087 |
| 8 | 0 | 0.0175 | **0.895** | 0 | 0.0218 | 0.0044 | 0.0175 | 0.0087 | 0.0087 | 0.0087 | 0.0175 | 0 |
| 9 | 0.0044 | 0.0349 | **0.777** | 0.0175 | 0.0131 | 0.0131 | 0.0175 | 0.0349 | 0 | 0.0437 | 0.0306 | 0.0131 |
| 10 | 0 | 0.0218 | 0.0568 | **0.852** | 0.0306 | 0.0131 | 0.0044 | 0.0044 | 0 | 0 | 0.0044 | 0.0131 |
| 11 | 0.0044 | 0.0349 | 0.0306 | **0.764** | 0.0175 | 0.0393 | 0.0087 | 0.0044 | 0.0175 | 0.0742 | 0.0044 | 0 |
| 12 | 0 | 0.048 | 0.048 | **0.738** | 0.0218 | 0.048 | 0.0131 | 0.0262 | 0.0175 | 0.0131 | 0.0087 | 0.0175 |
| 13 | 0.0175 | 0.0524 | 0.0742 | 0.0306 | **0.69** | 0.0131 | 0.0262 | 0.0437 | 0.0087 | 0.0218 | 0.0131 | 0.0087 |
| 14 | 0.0349 | 0.0437 | 0.0131 | 0.0087 | **0.668** | 0.083 | 0.0131 | 0.0349 | 0 | 0.0262 | 0.0393 | 0.0349 |
| 15 | 0.0131 | 0.0349 | 0.0175 | 0.0175 | **0.699** | 0.0437 | 0.0087 | 0.0349 | 0.0175 | 0.083 | 0.0218 | 0.0087 |
| 16 | 0.0175 | 0 | 0 | 0.083 | 0.0175 | **0.694** | 0.0044 | 0.0611 | 0.048 | 0.0568 | 0 | 0.0175 |
| 17 | 0.0262 | 0.0087 | 0.0044 | 0.1703 | 0.0349 | **0.686** | 0 | 0.0087 | 0.0393 | 0.0131 | 0.0044 | 0.0044 |
| 18 | 0.0175 | 0.0087 | 0 | 0.0087 | 0.0175 | **0.852** | 0 | 0.0306 | 0.0262 | 0.0087 | 0.0131 | 0.0175 |
| 19 | 0.0131 | 0.0131 | 0 | 0.0524 | 0.0044 | 0.0262 | **0.681** | 0.048 | 0.0175 | 0.1048 | 0.0175 | 0.0218 |
| 20 | 0.0087 | 0.0175 | 0.0087 | 0.0349 | 0.0131 | 0.0044 | **0.79** | 0.0175 | 0.0437 | 0.0218 | 0.0306 | 0.0087 |
| 21 | 0 | 0.0087 | 0 | 0.0131 | 0.0044 | 0.0175 | **0.886** | 0.0306 | 0.0175 | 0.0044 | 0.0044 | 0.0131 |
| 22 | 0.0044 | 0.0087 | 0.0437 | 0 | 0.0349 | 0.0655 | 0.0262 | **0.729** | 0 | 0.0699 | 0.0044 | 0.0131 |
| 23 | 0.0044 | 0.0044 | 0.0349 | 0.0044 | 0.0524 | 0.0393 | 0.0131 | **0.821** | 0.0044 | 0.0087 | 0.0044 | 0.0087 |
| 24 | 0.0393 | 0.0349 | 0.0175 | 0.0087 | 0.0699 | 0.1572 | 0.0393 | **0.594** | 0 | 0.0131 | 0.0131 | 0.0131 |
| 25 | 0 | 0.0044 | 0 | 0 | 0 | 0.0131 | 0.0131 | 0.0131 | **0.93** | 0.0087 | 0.0131 | 0.0044 |
| 26 | 0.0044 | 0 | 0.0044 | 0 | 0.0087 | 0.0044 | 0.0131 | 0.0087 | **0.93** | 0.0131 | 0.0131 | 0 |
| 27 | 0.0044 | 0.0131 | 0.0087 | 0 | 0.0218 | 0.0087 | 0.0218 | 0.0131 | **0.873** | 0.0131 | 0.0131 | 0.0087 |
| 28 | 0.0393 | 0.0349 | 0.0131 | 0.0087 | 0.0699 | 0.0524 | 0.0262 | 0.0175 | 0.1179 | **0.546** | 0.048 | 0.0262 |
| 29 | 0.0131 | 0.0175 | 0.0175 | 0 | 0.048 | 0.0087 | 0.0131 | 0.0087 | 0.0087 | **0.852** | 0.0044 | 0.0087 |
| 30 | 0.0175 | 0 | 0.0131 | 0.0087 | 0.0262 | 0.0306 | 0.0175 | 0.0044 | 0.0131 | **0.852** | 0.0131 | 0.0044 |
| 31 | 0.0087 | 0.0306 | 0.0306 | 0.0087 | 0.0087 | 0.0044 | 0.0131 | 0.0044 | 0.0044 | 0 | **0.878** | 0.0087 |
| 32 | 0.0175 | 0.0087 | 0.0218 | 0.0044 | 0.0218 | 0.0087 | 0.0044 | 0.0044 | 0.0087 | 0 | **0.878** | 0.0218 |
| 33 | 0.0349 | 0.0087 | 0 | 0.048 | 0.0044 | 0.0393 | 0.0393 | 0.0306 | 0.0044 | 0.0131 | **0.755** | 0.0218 |
| 34 | 0.0131 | 0.0306 | 0.1004 | 0.0218 | 0.0349 | 0.0218 | 0.0131 | 0.0218 | 0.0087 | 0.0961 | 0.0611 | **0.576** |
| 35 | 0.0306 | 0.0349 | 0.0655 | 0.0306 | 0.0131 | 0.048 | 0.0131 | 0.0961 | 0.0044 | 0.1135 | 0.0218 | **0.528** |
| 36 | 0.0742 | 0.0524 | 0.0655 | 0.0131 | 0.0437 | 0 | 0.0655 | 0.0175 | 0.0524 | 0.0917 | 0.0131 | **0.511** |

**Figure S1** Original plant material and their commercial form (Fengdou) of *Dendrobium* species using *D. officinale* as an example.

A-Cultivated *D. officinale*; B-Stacking from the original plant; C-Fresh stems for consume; D_1_-Commerical form (Tiepifengdou); D_2_-Juice product in daily life of Chinese.


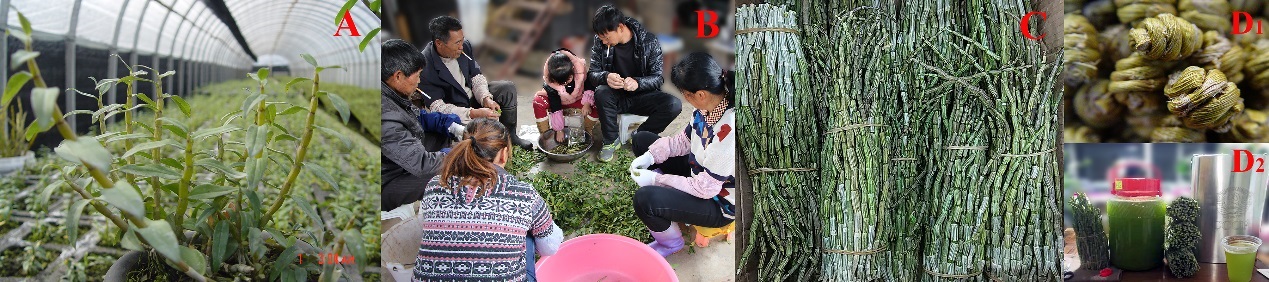


Figure S2 Score plots of PCA for 12 *Dendrobium* species using FTIR spectra after pretreatment. (A-PCA containing 12 categories; B-PCA containing two categories).


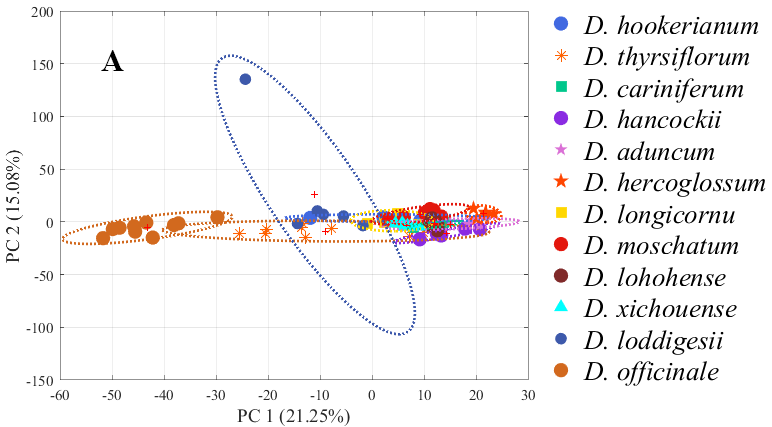

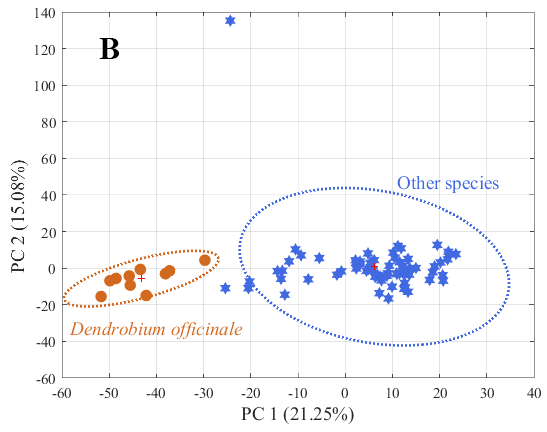


Figure S3 Original FTIR spectra of 12 *Dendrobium* species.


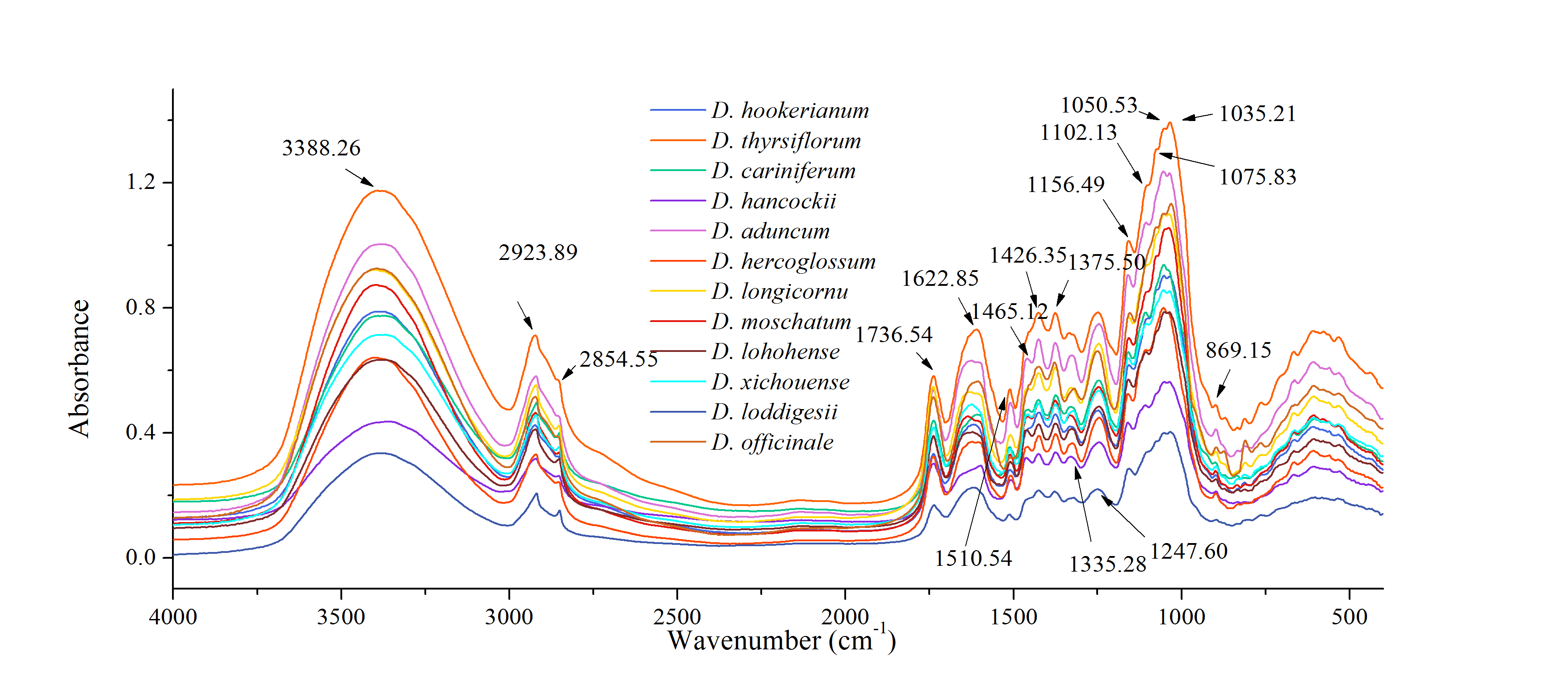

Supplement: Supplementary data and figure [file rsos190399supp1.docx]
